# Inactivation of the *FLCN* Tumor Suppressor Gene Induces TFE3 Transcriptional Activity by Increasing Its Nuclear Localization

**DOI:** 10.1371/journal.pone.0015793

**Published:** 2010-12-29

**Authors:** Seung-Beom Hong, HyoungBin Oh, Vladimir A. Valera, Masaya Baba, Laura S. Schmidt, W. Marston Linehan

**Affiliations:** 1 Urologic Oncology Branch, Center for Cancer Research, National Cancer Institute, Bethesda, Maryland, United States of America; 2 Basic Science Program, SAIC-Frederick, Inc., National Cancer Institute at Frederick, Frederick, Maryland, United States of America; Texas A&M University, United States of America

## Abstract

**Background:**

Germline mutations in a tumor suppressor gene *FLCN* lead to development of fibrofolliculomas, lung cysts and renal cell carcinoma (RCC) in Birt-Hogg-Dubé syndrome. TFE3 is a member of the MiTF/TFE transcription factor family and Xp11.2 translocations found in sporadic RCC involving *TFE3* result in gene fusions and overexpression of chimeric fusion proteins that retain the C-terminal DNA binding domain of TFE3. We found that GPNMB expression, which is regulated by MiTF, was greatly elevated in renal cancer cells harboring either *TFE3* translocations or *FLCN* inactivation. Since TFE3 is implicated in RCC, we hypothesized that elevated GPNMB expression was due to increased TFE3 activity resulting from the inactivation of *FLCN*.

**Methodology/Principal Findings:**

TFE3 knockdown reduced GPNMB expression in renal cancer cells harboring either *TFE3* translocations or *FLCN* inactivation. Moreover, FLCN knockdown induced GPNMB expression in *FLCN*-restored renal cancer cells. Conversely, wildtype FLCN suppressed GPNMB expression in *FLCN*-null cells. *FLCN* inactivation was correlated with increased TFE3 transcriptional activity accompanied by its nuclear localization as revealed by elevated GPNMB mRNA and protein expression, and predominantly nuclear immunostaining of TFE3 in renal cancer cells, mouse embryo fibroblast cells, mouse kidneys and mouse and human renal tumors. Nuclear localization of TFE3 was associated with TFE3 post-translational modifications including decreased phosphorylation.

**Conclusions/Significance:**

Increased TFE3 activity is a downstream event induced by *FLCN* inactivation and is likely to be important for renal tumor development. This study provides an important novel mechanism for induction of TFE3 activity in addition to TFE3 overexpression resulting from Xp11.2 translocations, suggesting that TFE3 may be more broadly involved in tumorigenesis.

## Introduction

Genetic studies have revealed several tumor suppressor genes [*von hippel lindau (VHL), fumarate hydratase (FH), succinate dehydrogenase subunit B (SDHB)* and *folliculin (FLCN)*] and proto-oncogenes (*hepatocyte growth factor receptor MET*) that are responsible for the development of renal cell carcinoma (RCC) [1]. In addition, two groups of transcription factors have been implicated in the development of RCC. Hypoxia-inducible factors (HIFs) were originally reported to be stabilized by the inactivation of the *VHL* tumor suppressor gene [2]. HIF stabilization is also correlated with mutations in *FH* or *SDHB* and is important in tumor cell growth and angiogenesis [3], [4]. TFE3 and TFEB, members of the MiTF/TFE transcription factor family, are highly expressed in the nucleus as a result of chromosomal translocations and are responsible for the development of juvenile renal cancer [5], [6]. However, the dysregulation of TFE3 or TFEB as a consequence of mutations in other tumor suppressor genes has not been reported. Here we investigated the regulation of TFE3 activity by the *FLCN* tumor suppressor gene.

Translocation renal cell carcinomas (RCCs) are rare tumors mainly reported in children and young adults [7]. They are classified as a distinct subtype and are characterized by various translocations that frequently involve TFE3 and, infrequently, TFEB. Both TFE3 and TFEB harbor basic helix-loop-helix-leucine zipper (bHLH-LZ) DNA binding domains and belong to the MiTF/TFE transcription factor subfamily, which also include microphthalmia transcription factor (MiTF) and transcription factor EC (TFEC) [8]. At least 5 genes have been reported to fuse with TFE3 and are predicted to produce ASPL-TFE3, PRCC-TFE3, NonO-TFE3, CLTC-TFE3 and PSF-TFE3 fusion proteins [7]. Importantly, the translocations are associated with overexpression of the fusion proteins, which can be identified by distinctive nuclear TFE3 staining. In addition, the resulting fusion proteins retain the bHLH-LZ DNA binding domain of TFE3, which may be important for tumorigenesis.

MiTF/TFE transcription factors bind to consensus M-box sequences (TCATGTG, CATGTGA or TCATGTGA) or the E-box sequence (CACGTG) either as homodimers or heterodimers [8], [9]. MiTF is a master regulator of melanocyte development and a key transcription factor in melanoma progression. It regulates genes involved in melanocyte survival and function, and melanoma cell proliferation. MiTF is often amplified in advanced melanomas and functions as an oncogene [10]. MiTF/TFE transcription factors show functionally redundant oncogenic activity. MiTF knockdown decreased viability of clear cell sarcoma cells but TFE3 or TFEB expression rescued their viability [11]. Reciprocally, TFE3 knockdown decreased viability of papillary renal carcinoma cells that harbor *TFE3* translocations but were rescued by expression of MITF.

GPNMB was first cloned from a melanoma cell line and is expressed at high level in many melanoma cell lines. Its expression is directly regulated by MiTF through highly conserved M-box sequences in the promoter [12], [13]. GPNMB is a glycosylated transmembrane protein and plays a role in osteoblast and osteoclast differentiation, and cancer cell metastasis [14]. It has been suggested as a therapeutic target in melanoma, glioblastoma and breast cancer [15]–[17]. An antibody-drug conjugate, CR011-vcMMAE, targeting GPNMB effectively induced complete regression of xenograft tumors expressing GPNMB [15]. CR011-vcMMAE is currently under phase I and II trials as a treatment for melanoma and breast cancer [18].

Birt-Hogg-Dube' (BHD) syndrome is characterized by the development of fibrofolliculomas, lung cysts and renal carcinoma and caused by germline mutations in the *folliculin* (*FLCN*) gene [19]–[21]. FLCN forms a complex with novel folliculin-interacting proteins 1 and 2 (FNIP1 and FNIP2), and 5′-AMP-activated protein kinase (AMPK), a kinase that negatively regulates mammalian target of rapamycin (mTOR) [22], [23]. The functional role of FLCN has been suggested through monitoring mTOR signaling after *FLCN* inactivation in cells and tissues. However, conflicting mTOR signaling or S6 phosphorylation results were reported [24]–[28]. Therefore regulation of mTOR activities by FLCN could be context dependent. Thus far there are no evident downstream target molecules that are strictly regulated by FLCN.

Renal carcinoma development is correlated with *FLCN* inactivation caused by naturally occurring germline mutations in human BHD patients, the Nihon rat model (C insertion in exon 3 of rat *Flcn*), German Shepherd dogs resulting in renal cystadenocarcinoma and nodular dermatofibrosis (RCND; H255R mutation in canine *Flcn*) and by genetically manipulated deletions in mice [21], [24]–[30]. *Flcn* heterozygous knockout mice developed renal neoplasia and cysts as they aged, with concomitant loss of the wildtype copy of *Flcn* [26]. On the other hand, kidney specific (cadherin 16)-Cre (Ksp-Cre)-mediated *Flcn* inactivation induces renal cell hyperproliferation and a polycystic kidney phenotype in mice [24].

In this study, we identified GPNMB as a downstream target that was induced by *FLCN* inactivation. GPNMB expression was investigated in renal cancer cells, mouse embryo fibroblast cells, and mouse and human renal carcinomas under conditions of *FLCN* inactivation. In addition, we examined the relationship between the *FLCN* tumor suppressor gene and the proto-oncogene *TFE3*, using GPNMB expression as a surrogate marker.

## Results

### GPNMB expression was elevated by *FLCN* inactivation or MiTF/TFE3 expression

**Figure 1:**
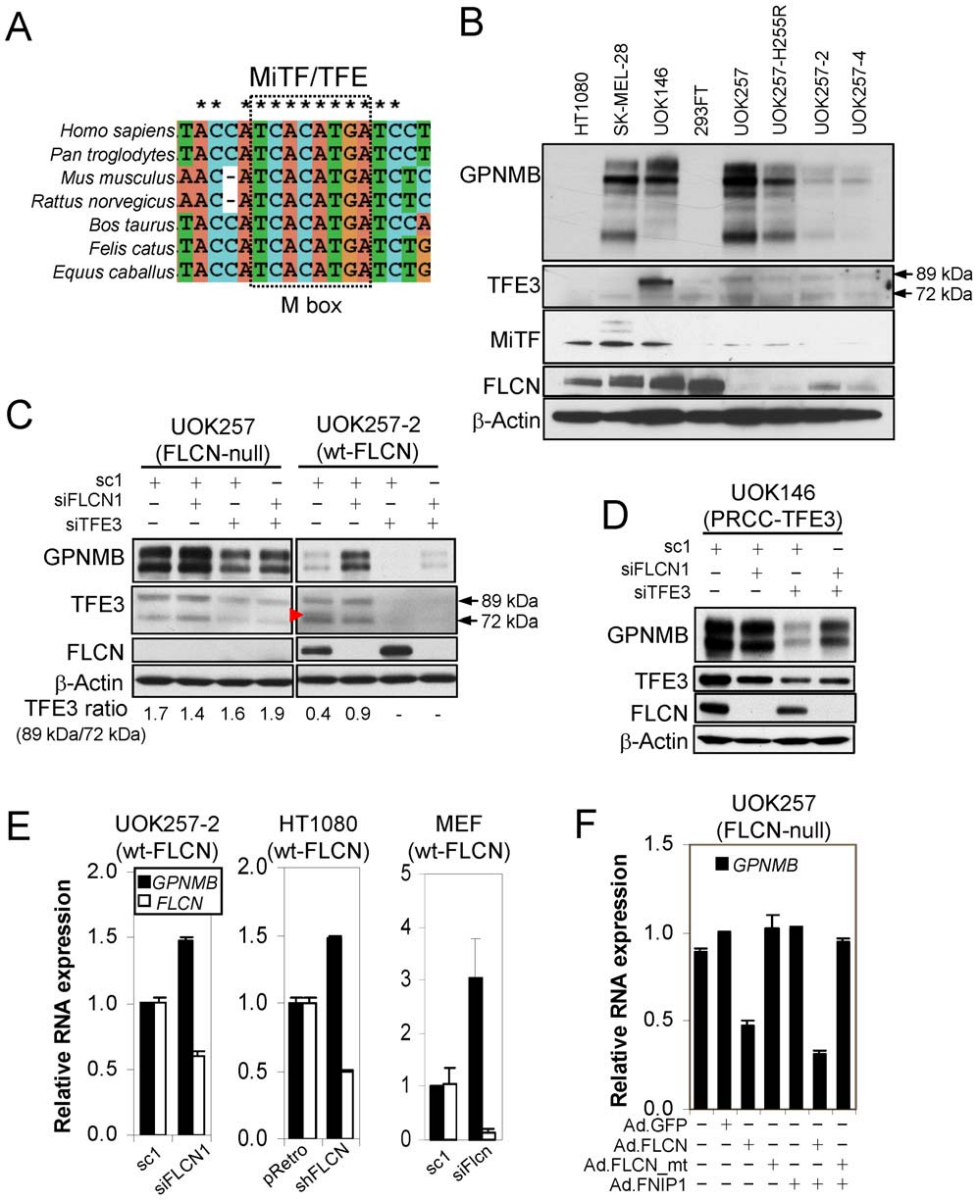
GPNMB expression was regulated positively by TFE3 and negatively by FLCN. (A) A highly conserved M box sequence (TCACATG) was identified in the *GPNMB* promoter that is recognized by the MITF/TFE3 transcription factor family. *GPNMB* promoter sequences from 8 representative mammalian species were obtained from the Ensembl database (www.ensembl.org) and were aligned for comparison using ClustalX (1.8) software (www.clustal.org). (B) GPNMB expression was examined in various cell lines by Western blot analysis. Two different TFE3 proteins (72 and 89 kDa) are indicated by arrows. (C–D) Regulation of GPNMB expression by TFE3 and FLCN knockdown. UOK257, UOK257-2 and UOK146 cells were transfected with control siRNA (sc1) or siRNAs targeting FLCN, TFE3 or FLCN/TFE3 and were harvested in 3 days for the analysis of protein expression. The ratios of TFE3^89 kDa^ to TFE3^72 kDa^ proteins are indicated. (E) FLCN knockdown induced GPNMB mRNA expression in UOK257-2, HT1080 and MEF cells. (F) Adenovirus-mediated FLCN or FLCN/FNIP1 overexpression suppressed GPNMB mRNA expression in UOK257 cells. GPNMB and FLCN expressions were analyzed by quantitative RT-PCR.

Previously in an effort to understand FLCN function, we searched for differentially expressed genes in cells expressing mutant FLCN compared to wildtype FLCN by gene expression microarray analysis. We identified ∼400 genes that were up or down-regulated more than 2-fold by the expression of wildtype FLCN [31]. Through a verification process including confirmation by RT-PCR and expression induction or reduction upon transient expression of FLCN, the number of genes for further analysis was reduced to 15 (Table S1). Twelve of 15 genes were upregulated and 3 of 15 genes were down regulated by adenoviral vector-mediated FLCN expression in UOK257 *FLCN*-null cells. We looked for a transcription factor that mediated this regulation. While evaluating the promoters of each gene by bioinformatics, we found that one of the 15 genes, *GPNMB*, is regulated by a transcription factor known as microphthalmia transcription factor (MiTF) [12], [13]. The *GPNMB* promoter harbors a highly conserved M box sequence, which is recognized by MiTF/TFE transcription factors (Fig. 1A). We examined whether MiTF and TFE3 transcription factor expressions were correlated with GPNMB expression (Fig. 1B). As in a previous report [32], we found high GPNMB expression in an MiTF-positive melanoma cell line, SK-MEL-28 (Fig. 1B). GPNMB expression was also high in the UOK146 cell line (Fig. 1B) that was established from a sporadic kidney tumor harboring a chromosomal translocation, t(X;1)(p11.2;q21), which expressed a high level of the resulting gene fusion product, PRCC-TFE3 [5]. Interestingly, although UOK257 cells expressed a high level of GPNMB similar to SK-MEL-28 and UOK146 cells, only a low level of MiTF was detected. On the other hand, a moderate level of two TFE3 isoforms, with molecular weights of 72 kDa and 89 kDa, were detected in UOK257 and its sublines, and in 293FT cells. The parental UOK257 cell line and a mutant *FLCN* (H255R) UOK257 cell line showed higher levels of GPNMB expression than the wildtype *FLCN-*restored cell lines (UOK257-2 and UOK257-4) (Fig. 1B). In accordance with GPNMB protein expression, GPNMB mRNA expression showed a similar pattern (Fig. S1A). However, MiTF and TFE3 levels were not affected by FLCN expression. HT1080 and 293FT cells expressed MiTF or TFE3 proteins at comparable levels to SK-MEL-28 and UOK257, respectively, but neither 293FT cells nor HT1080 cells expressed GPNMB (Fig. 1B). This suggested that MiTF and TFE3 may not be constitutively active but may need stimulation or additional factors to induce GPNMB expression.

### GPNMB expression was regulated negatively by FLCN and positively by TFE3

In order to examine whether TFE3 regulates GPNMB expression, TFE3 expression was knocked down by siRNA in UOK257 and UOK146 cells. TFE3 siRNAs reduced both 72 kDa and 89 kDa protein bands in UOK257 cells, which were recognized by TFE3 antibody, confirming that both proteins were TFE3. UOK146 expressed only one PRCC-TFE3 fusion protein migrating similarly to the larger TFE3 protein and its expression was reduced by TFE3 siRNA. Interestingly, TFE3 knockdown reduced GPNMB expression in both UOK257 and UOK146 cell lines (Fig. 1C and 1D). However, FLCN siRNA induced GPNMB expression only in *FLCN*-expressing cells (UOK257-2), but not in *FLCN*-null cells (UOK257; Fig. 1C). GPNMB expression was higher in UOK257-2 and UOK146 cells with FLCN and TFE3 double knockdown compared to those cells with TFE3 knockdown alone (Fig. 1C and 1D).

To examine whether GPNMB expression induced by *FLCN* inactivation was through transcriptional regulation, we measured GPNMB mRNA expression after FLCN knockdown by siRNAs or shRNA in the UOK257-2, HT1080 and MEF cell lines. When FLCN expression was reduced, GPNMB mRNA levels were increased 1.5∼3 fold compared to the controls (Fig. 1E). Conversely, adenovirus mediated wild-type FLCN or FLCN/FNIP1 expression suppressed GPNMB mRNA expression more than 2 fold, but mutant FLCN (c.1285dupC, most frequent mutation in BHD syndrome), mutant FLCN/FNIP1 or FNIP1 alone did not suppress GPNMB expression in UOK257 cells (Fig. 1F). In accordance with the GPNMB mRNA expression, the GPNMB protein level was also induced by FLCN knockdown and reduced by adenovirus mediated-wildtype FLCN expression but not by mutant FLCN expression (c.1285dupC) (Fig. S1B and S1C).

### *GPNMB* promoter activity was suppressed by FLCN or FLCN/FNIPs

**Figure 2:**
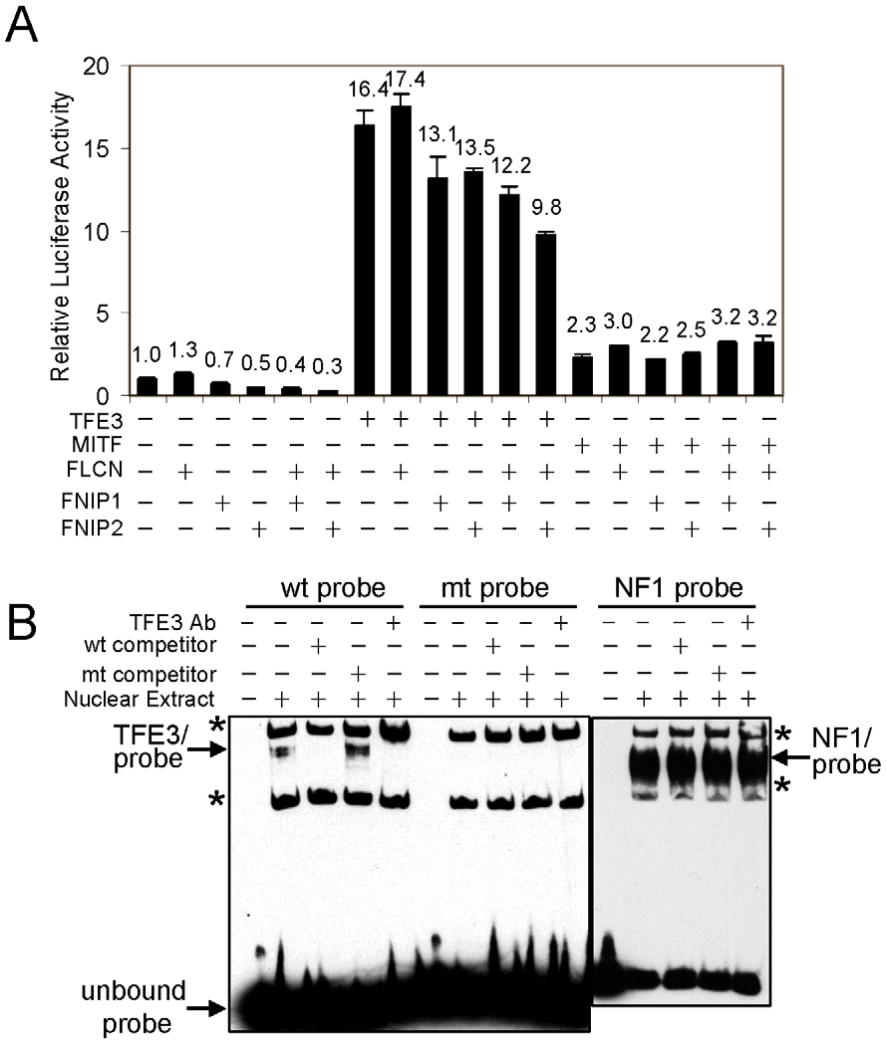
Regulation of TFE3-driven *GPNMB* promoter activity by FLCN and FNIP1/2. (A) FLCN/FNIP1/2 suppressed basal and TFE3-induced but not Mitf-induced *GPNMB* promoter activity. HT1080 cells were transfected with *GPNMB* promoter-luciferase-reporter construct along with FLCN, FLCN/FNIP1 or FLCN/FNIP2. Promoter activity was calculated after normalizing firefly luciferase reporter activity to *TK*-promoter driven renilla luciferase activity. (B) EMSA showed TFE3 bound to *GPNMB* promoter sequence containing wildtype M box (wt probe) but not mutant M box (mt probe). *, non-specific bands.

*GPNMB* promoter activity was analyzed in HT1080 cells that express endogenous FLCN. Basal *GPNMB* promoter activity was not reduced by ectopic expression of FLCN but was reduced by expression of FNIP1 (30%; student's t-test, P = 0.06) or FNIP2 (50%; P<0.01) and synergistically by FLCN/FNIP1 (60%; P<0.01) or FLCN/FNIP2 (70%; P<0.01) (Fig. 2A). Similarly, TFE3-induced *GPNMB* promoter activity was not suppressed by FLCN but was suppressed by FNIP1 (20%; P<0.05) or FNIP2 (20%; P<0.01) and further by FLCN/FNIP2 (40%; P<0.01) suggesting that both FLCN and FNIP are needed in the suppression of TFE3 activity (Fig. 2A). However, MiTF-induced *GPNMB* promoter activity was not reduced by FLCN or FLCN/FNIPs. Electrophoretic mobility shift assays (EMSA) confirmed that TFE3 bound to the *GPNMB* promoter sequence containing the wildtype M box but not a mutant M box (Fig. 2B). TFE3 binding to the *GPNMB* promoter sequence was validated by the disappearance of the probe/TFE3 band with TFE3 antibody possibly through a supershift of the band. Although we could not observe a discrete supershifted band, the increased intensity of the upper non-specific band by the addition of TFE3 antibody suggested a supershift of the TFE3/probe band.

### TFE3 post-translational modifications were affected by FLCN expression

**Figure 3:**
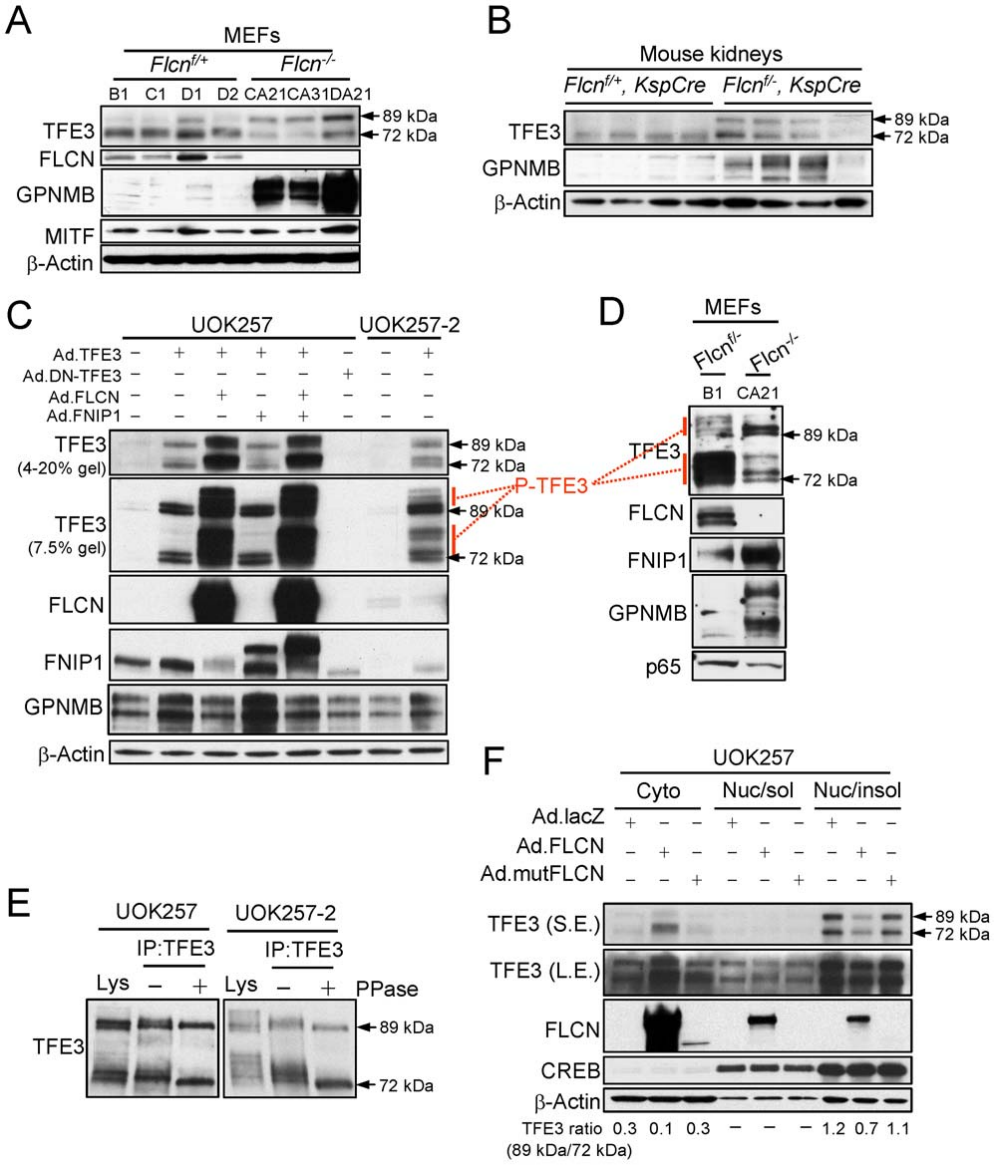
*FLCN* inactivation was correlated with post-translational modifications and nuclear accumulation of TFE3. (A–B) Increased Gpnmb expression and increased ratio of TFE3^89 kDa^ to TFE3^72 kDa^ was correlated with *Flcn* inactivation in MEFs and mouse kidneys. (C) Adenovirus- mediated FLCN expression increased phosphorylation of both TFE3^89 kDa^ and TFE3^72 kDa^ proteins in UOK257 cells. (D) Higher level of TFE3 phosphorylation was observed in *Flcn^f/−^* MEFs compared to *Flcn^−/−^* MEFs. (E) TFE3 phosphorylation was examined by treating TFE3 immunoprecipitates with or without protein phosphatase (PPase). Lys, cell lysate. (F) TFE3 subcellular localization was analyzed by fractionating cells into cytoplasmic (Cyto), soluble nuclear (Nuc/sol) and insoluble nuclear (Nuc/insol) fractions. The ratio of TFE3^89 kDa^ to TFE3^72 kDa^ was calculated from the band intensities of TFE3 by densitometry (S.E.). S.E., short exposure; L.E., long exposure.

Two different molecular weight TFE3 proteins (72 kDa and 89 kDa) were frequently observed in many cell types, including UOK257, 293FT, mouse embryonic fibroblasts (MEFs) and *Flcn* knockout mouse kidneys (Fig. 1B, 3A and 3B). In addition, both TFE3 proteins were produced in UOK257 from a single adenovirus-delivered full-length mouse TFE3 cDNA suggesting protein modification. Although the molecular weight of TFE3 is 62 kDa, it was reported that TFE3 protein from B16 melanoma cells and mouse splenocytes migrates close to 72 kDa [33]. Thus the lower 72 kDa protein can be regarded as the native form of TFE3 and designated as TFE3^72 kDa^. The upper 89 kDa TFE3 protein, designated as TFE3^89 kDa^ is likely to be a product of post-translational modification of TFE3. In both MEFs and mouse kidneys, *FLCN* inactivation was correlated with an increased level of TFE3^89 kDa^ compared to TFE3^72 kDa^, and increased GPNMB protein and mRNA expression (Fig. 3A, 3B, S1D and S1E). In addition, the ratio of TFE3^89 kDa^ to TFE3^72 kDa^ was lower in UOK257-2 (ratio = 0.4) compared to UOK257 (ratio = 1.7) but it was increased in response to FLCN knockdown by siRNA (ratio = 0.9) (Fig. 1C). Not only the ratio of TFE3^89 kDa^ to TFE3^72 kDa^, but also the migration pattern of TFE3^72 kDa^ was different between UOK257 and UOK257-2. The smeared additional band just above TFE3^72 kDa^ disappeared after FLCN knockdown (Fig. 1C, arrow head). To better examine the effect of FLCN expression on TFE3 post-translational modifications, FLCN and TFE3 were ectopically expressed in UOK257 cells using adenoviral vectors, and the cell lysates were separated by either 4–20% or 7.5% SDS PAGE (Fig. 3C). We could see doublet bands for TFE3^72 kDa^ and TFE3^89 kDa^ proteins in UOK257 cell lysates when separated by 7.5% SDS PAGE (Fig. 3C, lane 2). However, we could observe at least two or more slower migrating bands for each protein in UOK257-2 cells (Fig. 3C, lane 8, red vertical bars). In addition, ectopic expression of wildtype FLCN and FLCN/FNIP1 induced post-translational modifications in both TFE3 proteins in UOK257 cells (Fig. 3C, lanes 3 and 5). We then examined whether the multiple slower migrating TFE3 bands were due to phosphorylation. TFE3 proteins were immunoprecipitated from UOK257 and UOK257-2 cell lysates and were treated with protein phosphatase. As shown in Fig. 3E, the slower migrating smeared bands were down-shifted and became a single sharp band for each TFE3 protein, indicating that those slower migrating bands were phosphorylated TFE3 proteins. TFE3 phosphorylation was also examined in MEFs by separating cell lysates through 7.5% SDS PAGE. As shown in Fig. 3D, both TFE3^72 kDa^ and TFE3^89 kDa^ proteins were more phosphorylated in *Flcn* heterozygote MEFs compared to *Flcn*-inactivated MEFs similar to UOK257-2 and UOK257 (red vertical bars).

### FLCN induced cytoplasmic accumulation of TFE3

Transcription factor activity can be regulated by modulating subcellular localization, which is often achieved through post-translational modification including phosphorylation and dephosphorylation. Translocation of MiTF and TFEB into the nucleus in response to stimulus has been studied but nucleocytoplasmic shuttling of TFE3 has not been reported [34], [35]. Since TFE3 post-translational modifications were affected by FLCN, we examined whether FLCN expression could regulate TFE3 subcellular localization after cellular fractionation to yield cytoplasmic, soluble and insoluble nuclear fractions. Interestingly, endogenous TFE3 nuclear accumulation was negatively regulated by FLCN expression (Fig. 3F). TFE3 was mainly localized in the insoluble nuclear fraction of UOK257 cells. However, adenovirus-mediated FLCN expression increased TFE3 levels in the cytoplasmic fraction and decreased TFE3 levels in the insoluble nuclear fraction. The ratio of TFE3^89 kDa^ to TFE3^72 kDa^ was higher in the nuclear fraction (1.2) than in the cytoplasmic fraction (0.3) in UOK257 cells; however, the ratio was reduced in both fractions by wildtype but not mutant FLCN (c.1285dupC) expression (Fig. 3F). Nonetheless the degree of reduction was greater in the cytoplasmic fraction (0.3 = >0.1, 67%) than in the nuclear fraction (1.2 = >0.7, 42%).

**Figure 4:**
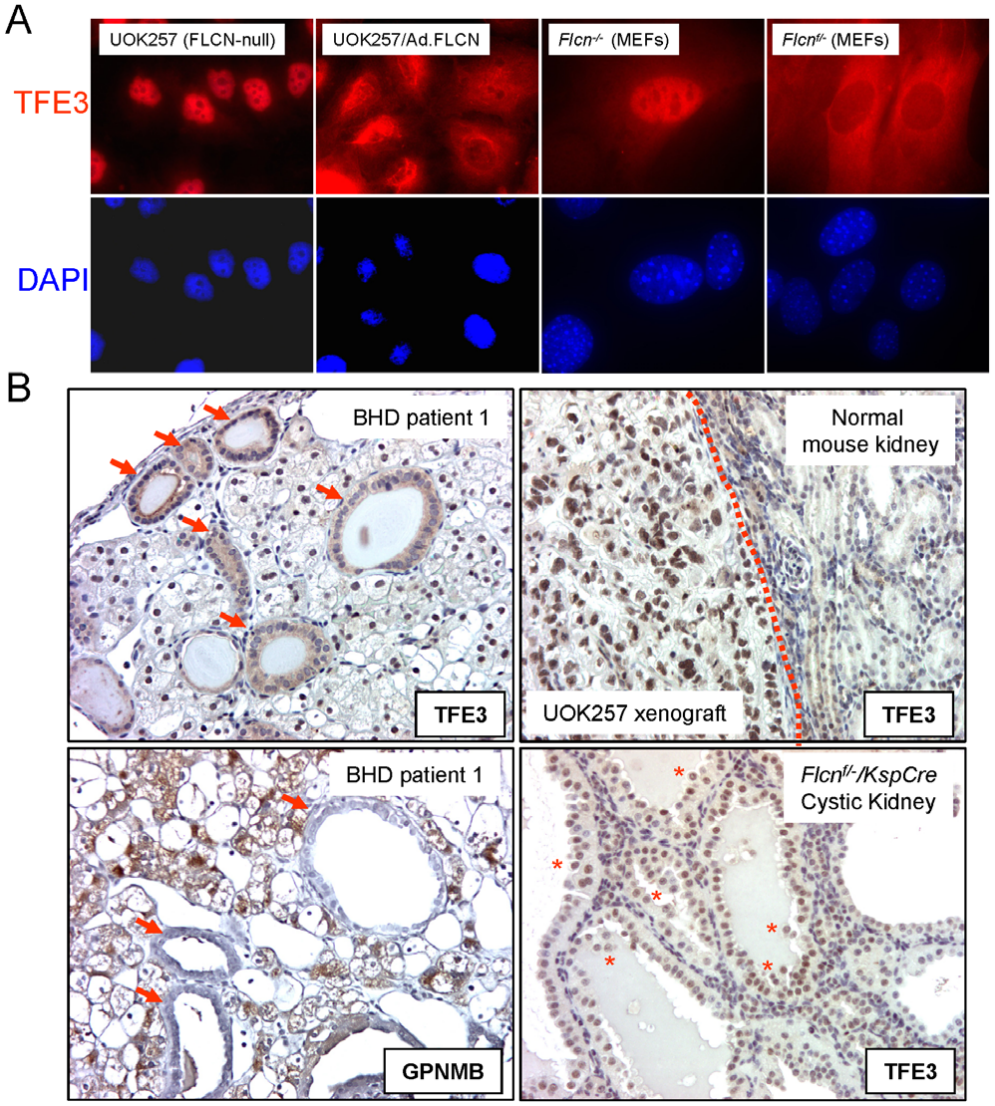
Nuclear localization of TFE3 in *FLCN* inactivated cells. (A) Cells were plated on chamber slides and transduced with adenovirus expressing TFE3 alone or with FLCN. TFE3 proteins were probed with rabbit polyclonal TFE3 antibody (1∶450 dilutions) as described in Materials and Methods. Nuclei were counter-stained with DAPI. (B) Immunohistochemical staining of TFE3 or GPNMB in the renal tumors from BHD patients, and immunostaining of TFE3 in the UOK257 xenograft tumors from nude mice and in the cystic kidneys of *Flcn^f/−^/KspCre* mice. Arrows, the normal renal tubules imbedded in tumor cells. Asterisks (*), the renal epithelial cells detaching from tubules.

In accordance with the fractionation result, immunocytochemical staining of adenovirus-delivered TFE3 proteins showed nuclear localization of TFE3 in UOK257 cells (Fig. 4A). Transient FLCN expression induced cytoplasmic accumulation of TFE3 proteins in UOK257 cells. In addition, we observed that not only adenovirus-delivered TFE3 but also endogenous TFE3 proteins were localized in the nucleus of *Flcn-*null (*Flcn ^−/−^*) MEFs, whereas TFE3 was localized in the cytoplasm of the *Flcn* heterozygote (*Flcn ^f/−^*) MEFs (Fig. 4A and S2A). Immunohistochemical staining exhibited nuclear or nuclear/cytoplasmic TFE3 staining in the tumor cells from BHD patients (Fig. 4B and S2B) although the intensity was not as strong as in the t(X;1)(p11.2;q21) translocation alveolar soft part sarcoma involving TFE3 (Fig. S2B). Diffused cytoplasmic TFE3 staining was observed predominantly in the embedded normal renal tubules and in the normal kidney adjacent to tumor although some cytoplasmic/nuclear staining was also observed (Fig. 4B, top left). In accordance with the TFE3 staining pattern, GPNMB expression was restricted to renal tumor cells and was absent from embedded normal renal tubules (Fig. 4B, bottom left). Nuclear TFE3 staining was observed in the cystic mouse kidneys resulting from Ksp-Cre-mediated *Flcn* inactivation (Fig. 4B, bottom right). UOK257 xenograft tumors showed strong nuclear TFE3 staining whereas the adjacent mouse kidneys showed weak and diffused cytoplasmic or cytoplasmic/nuclear TFE3 staining (Fig. 4B, top right).

### GPNMB expression was high in renal tumors from BHD patients and a *Flcn^+/−^* heterozygous knockout mouse model

**Figure 5:**
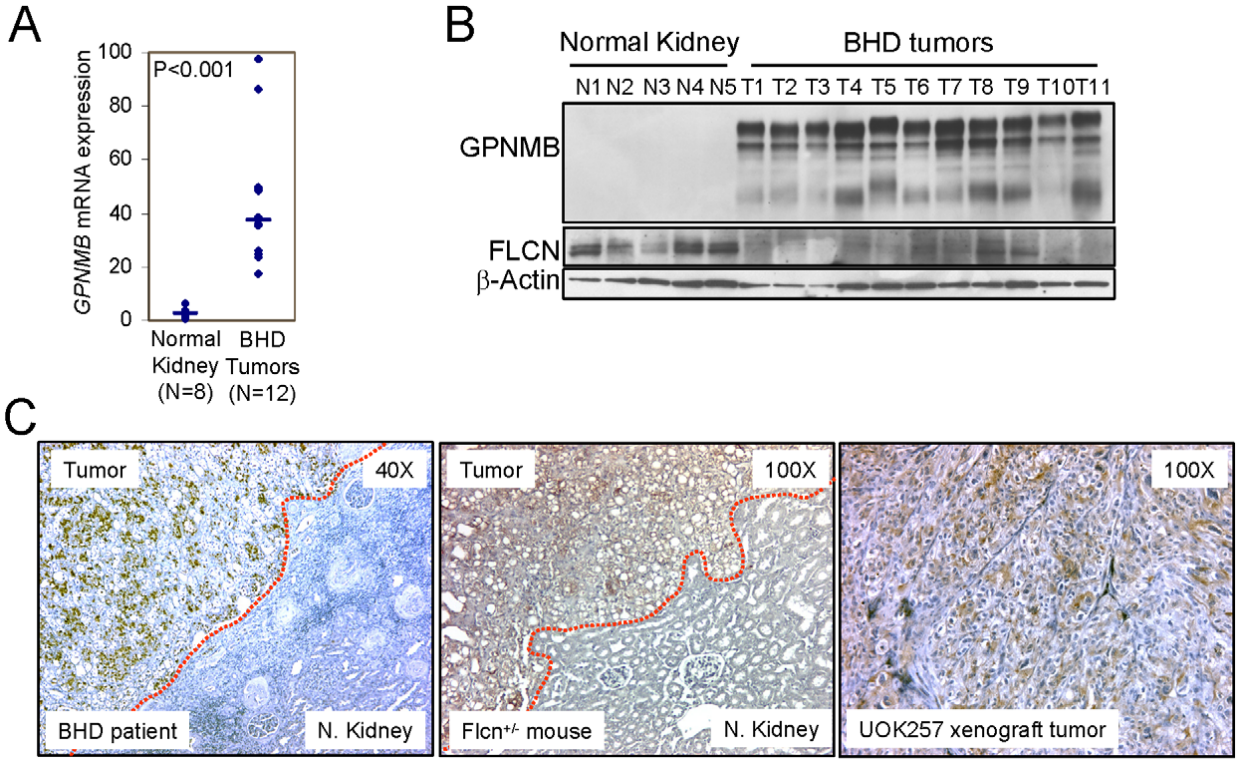
High level of GPNMB expression in the renal tumors from BHD patients and *Flcn* heterozygous knockout mice. (A) GPNMB mRNA and (B) protein expressions were measured by quantitative RT-PCR and Western blotting, respectively in the renal tumors and normal kidneys. (C) Immunohistochemical staining of GPNMB in the tumors adjacent to normal kidneys from BHD patients or *Flcn*-heterozygous knockout mice. GPNMB staining in the UOK257 xenograft tumors grown in nude mice.

We examined GPNMB expression as a readout of TFE3 transcriptional activity in the renal tumors from BHD patients. All of the tumors (N = 14) from BHD patients expressed 40-fold higher levels of GPNMB mRNA on average (P<0.001) compared to normal kidney tissues (N = 8) (Fig. 5A). Western blots also showed high levels of GPNMB protein expression in all of the tumors from BHD patients (N = 11) but undetectable levels of GPNMB expression in all normal kidney tissues (N = 5) (Fig. 5B). Immunohistochemical staining further confirmed that GPNMB expression was exclusively located in the tumor portion but not in the normal kidney portion of sections from BHD patients and *Flcn^+/−^* heterozygote mouse renal tumors (Fig. 5C). The UOK257 xenograft tumors were also strongly positive for GPNMB staining (Fig. 5C). These results showed that TFE3 transcriptional activity was elevated in renal tumors with *FLCN* inactivation.

### Identification of the downstream target genes regulated by TFE3 and FLCN

**Figure 6:**
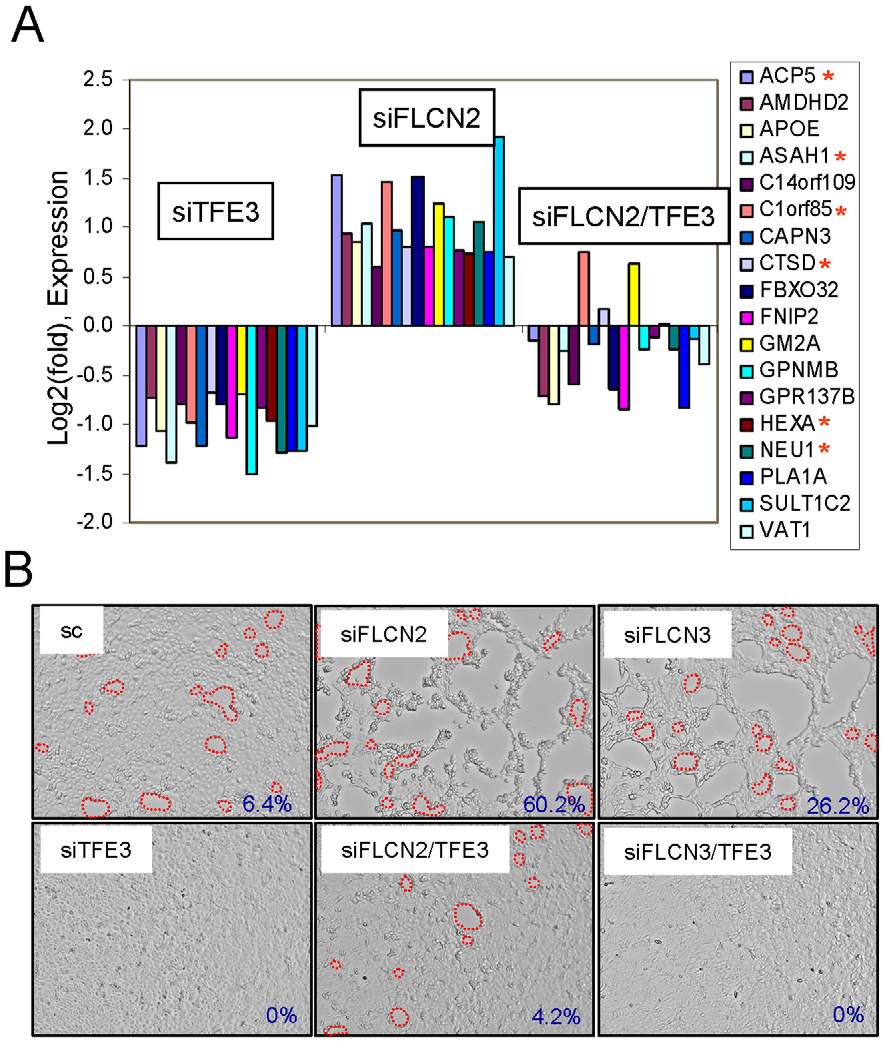
The genes and the biological processes positively regulated by TFE3 and negatively regulated by FLCN. (A) Identification of TFE3 downstream target genes that were positively regulated by FLCN knockdown. UOK257-2 cells were transfected with siRNAs targeting FLCN, TFE3 or FLCN/TFE3. Gene expressions were examined with Human Genome U133 Plus 2.0 Array. A subset of genes that were commonly regulated by MiTF/TFE transcription factors and by FLCN are shown in the graph. *, lysosomal genes. (B) UOK146 cell detachment was enhanced by FLCN knockdown but suppressed by TFE3 knockdown. UOK146 cells were transfected with siRNAs targeting FLCN and/or TFE3, and cell morphology was photographed 30 hrs after transfection. Boundaries of detached areas that are difficult to see are denoted by dotted red lines. Detached areas were quantitated using ImageJ software (NIH, USA) and denoted as a percentage on the lower right corner of each figure.

We wanted to find the downstream target genes of TFE3 other than GPNMB, which are also dysregulated by the inactivation of *FLCN*. In order to find these genes, we performed microarray analysis of UOK257-2 cells after siRNA knockdown of TFE3 and FLCN independently or together. We identified ∼110 genes that were regulated positively more than 1.5 fold by FLCN knockdown and negatively by TFE3 knockdown (Table S2). In order to identify the genes that were directly regulated by TFE3, we examined the gene promoters for MiTF/TFE recognition sequences using the MatInspector program (www.genomatix.de). We found 48 genes that have one or more MiTF/TFE binding site(s) in their promoters (Table S3). We compared those genes identified by microarray with the reported MiTF and TFEB targets [36], [35]. Eighteen of them were among the reported targets of either MiTF or TFEB (Fig. 6A). Interestingly, 6 lysosomal genes and the FLCN interacting protein FNIP2 were regulated by TFE3. The expression changes of two representative lysosomal genes (*ACP5* and *ASAH1*), *FNIP2* and *SULT1C2* were confirmed by RT-PCR following TFE3, FLCN or TFE3/FLCN knockdown in UOK257-2 cells (Fig. S3).

### Increased TFE3 dependent cell detachment by FLCN knockdown

UOK146 cells expressed a high level of PRCC-TFE3 fusion protein and grew on culture dishes with typical morphological characteristics. We observed a few detaching cells and circular areas devoid of cells during cell culture (Fig. 6B, top left), which were dependent on TFE3 expression since these phenotypes were completely abrogated by TFE3 knockdown (Fig. 6B, bottom left). Cell detachment was greatly increased by FLCN knockdown with two independent siRNAs leaving isolated clumps of cells loosely attached to the dish (Fig. 6B, upper panels, middle and right). Cell detachment began about 48 hrs after siRNA transfection and became more severe as time progressed. Conversely, cell detachment by FLCN knockdown was dramatically attenuated by TFE3 knockdown (Fig. 6B, lower panels, middle and right).

## Discussion

In this study, in an effort to elucidate FLCN function we sought to find downstream target genes that were regulated by FLCN and the transcription factors that mediated such regulation. We initially found that GPNMB expression was strictly dependent on *FLCN* inactivation in most of the cell types we examined. Then we tested whether GPNMB expression could be regulated by TFE3 since the *GPNMB* promoter contained a highly conserved M box sequence that could be recognized by MiTF/TFE transcription factors. Our results showed that GPNMB expression was regulated by TFE3 and that TFE3 activity was induced through its nuclear localization as a result of *FLCN* inactivation. TFE3 nuclear localization was correlated with reduced phosphorylation of TFE3. Here we provide evidence for a new signaling pathway that leads to the activation of an oncogenic transcription factor TFE3 by a mechanism that is different from Xp11.2 translocations. This is the first report connecting inactivation of a tumor suppressor *FLCN* to activation of an oncogenic transcription factor TFE3, both of which are important in the development of RCC.

This study demonstrates the usefulness of GPNMB as a surrogate marker for TFE3 activity and *FLCN* inactivation. GPNMB expression was highly dependent on *FLCN* inactivation not only in cultured cells but also in the kidneys and renal tumors of the human patient and *in vivo* mouse models. Specifically, high levels of GPNMB expression were found in all of the renal tumors (N = 11) from BHD patients, but none of the normal kidney tissues (N = 5), suggesting that GPNMB might serve as a biomarker for BHD syndrome and a therapeutic target for treatment of BHD renal tumors (Fig. 5). GPNMB is highly expressed in several tumor types such as melanoma, glioblastoma and breast cancer [15]–[17]. Since MiTF is often amplified in melanoma, GPNMB expression in melanoma cells is likely to be a result of elevated MiTF transcriptional activity. However, the transcription factor that is responsible for the GPNMB expression in glioblastoma and breast cancer has not been investigated. Thus, it would be interesting to investigate whether FLCN and TFE3 are involved in the regulation of GPNMB in those tumors. We are also considering the possibility that an antibody-drug conjugate targeting GPNMB might be useful as a therapeutic intervention in BHD patients.

In addition to *GPNMB*, we have found through microarray and promoter sequence analysis at least 47 more potential downstream targets of TFE3 that have MiTF/TFE binding sites in their promoter regions (Table S3). These genes may be important in TFE3-mediated tumorigenesis and tumor cell survival and proliferation. From among the 48 genes, we have identified 18 genes that were known targets of MiTF and/or TFEB, suggesting that they are commonly regulated by MiTF/TFE transcription factors (Fig. 6A). Interestingly, *FNIP2* was shown to be regulated by both TFEB and TFE3, and its promoter harbors a highly conserved M box sequence (Fig. S4). Since expression of FLCN/FNIP2 suppresses TFE3 activity, TFE3 mediated FNIP2 induction could be a negative feedback mechanism. Further study of FNIP2 function and expression is needed to reveal the details of such a mechanism.

A recent report suggested an important role for TFEB in the regulation of lysosome biogenesis and function through its binding to CLEAR elements (GTCACGTGAC) in the promoters of many lysosomal genes [35]. The CLEAR consensus sequence overlaps the E-box sequence (CACGTG) and is similar to the MiTF/TFE consensus sequence (TCACATGA). Several lysosomal genes (*ACP5*, *ASAH1*, *NEU1*, *HEXA*, *C1orf85* and *CTSD*) that were shown to be regulated by TFEB were also regulated by TFE3 and FLCN (Fig. 6A). Thus it would be interesting to examine whether *FLCN* inactivation and concomitant TFE3 activation affect lysosomal biogenesis and function through the transcriptional regulation of those lysosomal genes.

Translocation-induced overexpression of TFE3 or TFEB fusion proteins is important in proliferation, anchorage independent growth, migration and long term survival of cancer cells [37], [11]. Since the fusion proteins strongly stained in the nucleus and retain DNA binding domains, it is likely that nuclear accumulation and transcriptional activities of TFE3 and TFEB are indispensable for their tumorigenic activity. In support of this idea, ectopic expression of wild-type, unfused TFE3 stimulates anchorage independent tumor cell growth [6]. In addition, Alpha-TFEB gene fusions were found in primary renal tumors, which could result in the expression of intact TFEB proteins through strong *Alpha* gene promoter activity. This would suggest that dysregulated expression, rather than altered function of TFE3 and TFEB fusion proteins, may confer the tumorigenic potential of TFE3 and TFEB [6]. Although TFE3 protein expression was not elevated, TFE3 transcriptional activity, as revealed by GPNMB expression, was greatly induced by *FLCN* inactivation. Thus it is likely that elevated TFE3 transcriptional activity as a consequence of *FLCN* inactivation contributes to the development of renal carcinoma.

Nucleocytoplasmic shuttling is one of the major mechanisms in the regulation of transcription factors including MiTF and TFEB [38], [35]. However, the regulation of TFE3 nucleocytoplasmic shuttling has not been described. This study is the first report showing the regulation of TFE3 nucleocytoplasmic shuttling. We have shown that nuclear localization of TFE3 was correlated with TFE3 post-translational modifications including decreased phosphorylation and an undetermined modification that induces accumulation of TFE3^89 kDa^ over TFE3^72 kDa^. Transcription factors can be either imported into the nucleus or exported to the cytoplasm depending on the stimulus. Nucleocytoplasmic shuttling of transcription factors are often accompanied by post-translational modifications including phosphorylation/dephosphorylation, sumoylation, and ubiquitination. Although TFE3 phosphorylation and sumoylation are reported, their relevance to nucleocytoplasmic shuttling has not yet been investigated [39], [40]. Thus an in-depth investigation of the relationship between the post-translational modifications and the nucleocytoplasmic shuttling of TFE3 would potentially reveal the mechanism by which *FLCN* inactivation regulates TFE3 activity.

Our current research effort is directed toward finding an answer to the question of how FLCN regulates TFE3 post-translational modifications. In order to answer that question, future experiments will examine kinases/phosphatases that regulate TFE3 phosphorylation/dephosphorylation and the undetermined post-translational modification that increases accumulation of TFE3^89 kDa^. In addition, it will be important to investigate the possible involvement of FLCN, FNIP1/2 and AMPK in the regulation of TFE3. Our current data suggested that FLCN and FNIP1/2 suppress TFE3 transcriptional activity synergistically. We showed that ectopic FLCN expression did not suppress *GPNMB* promoter activity in FLCN-wildtype HT1080 cells (Fig. 2A). However, ectopic expression of FNIP1 suppressed *GPNMB* promoter activity in HT1080. In addition both FLCN/FNIP1 and FLCN/FNIP2 suppressed basal and TFE3-induced *GPNMB* promoter activity in HT1080 cells. An important question remains as to whether AMPK is involved in the regulation of TFE3. It has been reported that TFE3 induces the expression of metabolic genes such as *IRS2*, *HK2* and *INSIG1*, resulting in glucose uptake, glycogen synthesis and protein synthesis in the liver [41]. Since AMPK is a kinase that is activated in cells with low energy and regulates cellular proteins that are involved in energy metabolism, it could be possible that AMPK regulates TFE3 directly or indirectly through FLCN/FNIP or under the regulation of FLCN/FNIP. TFE3 post-translational modifications and its subcellular localization could be an important readout for the analysis of FLCN function and the function of the FLCN/FNIP1/FNIP2/AMPK complex. Further study will clarify the functional relationship between FLCN, FNIP1, FNIP2 and AMPK.

In conclusion, we have identified a specific member of the MiTF/TFE family of transcription factors, the oncogenic transcription factor TFE3, that was regulated by the inactivation of the *FLCN* tumor suppressor gene through induction of TFE3 nuclear localization. TFE3 nuclear localization was correlated with decreased phosphorylation and increased accumulation of TFE3^89 kDa^ over TFE3^72 kDa^. We characterized GPNMB as a downstream target of TFE3, whose expression was strictly dependent on *FLCN* inactivation in cultured cells, kidneys of *Flcn* knockout mouse models, and kidney tumors from BHD patients (a working model is shown in Fig. S5). This study will shed light on the understanding of FLCN/FNIP1/FNIP2/AMPK function and the downstream target genes and signaling pathways that are important in tumorigenesis, providing insight into therapeutic targets for treatment of renal tumors that develop in BHD syndrome and translocation RCC.

## Materials and Methods

### Cell culture, reagents and transfections

UOK257 cells harboring mutation in *FLCN* (c.1285dupC) and their subclones expressing either wildtype (UOK257-2 and UOK257-4) or mutant FLCN (UOK257-H255R) are described [22], [31], [42]. *Flcn* heterozygous knockout MEFs (*Flcn* ^f/−^) were generated from E13.5 mouse embryos harboring floxed and deleted *Flcn* alleles [32]. *Flcn*-null MEFs (*Flcn* ^−/−^) were produced from *Flcn* ^f/−^ MEFs by adenovirus expressing Cre recombinase. *Flcn* ^f/−^ (B1, C1, D1 and D2) and *Flcn* ^−/−^ (CA21, CA31 and DA21) MEF clones were obtained through spontaneous immortalization. UOK146 cells containing the translocation that results in the fusion of PRCC at 1q21.2 to the TFE3 gene at Xp11.2 are described [5]. Cells were cultured in DMEM medium supplemented with 10% fetal bovine serum (FBS) and penicillin/streptomycin. Cells were transfected with siRNAs using Dharmafect 4 (Dharmacon) according to the manufacturer's protocol. The following siRNAs were used for transfection. Human FLCN siRNA (siFLCN1, Thermo Scientific, J-009998-05-0005); Non-targeting siRNA pool (sc1, Thermo Scientific, D-001810-10-20); Stealth siRNAs targeting FLCN (Invitrogen): siFLCN2 (5′- CACCCGGGAUAUAUCAGCCAUGAUA-3′), siFLCN3 (5′- CAAGGUGUUUGAGGCAGAGCAGUUU-3′); Stealth non-targeting scrambled siRNAs (Invitrogen): sc2 (5′-CACGGGAUAUAUGACACCGUCCAUA-3′), sc3 (5′- CAAUUGUGAGUGACGACGAGGGUUU-3′); Human TFE3 siRNA pool (siTFE3, Thermo Scientific, L-009363-00-0005); Mouse Flcn siRNA pool (siFlcn, Thermo Scientific, L-050651-01-0005). HT-1080 cells were infected with the retroviruses expressing FLCN shRNA [31], and selected against puromycin (2.5 µg/ml). Adenoviruses expressing wildtype or mutant (c.1285dupC) FLCN have been described [31]. Adenoviruses expressing wildtype or dominant negative TFE3 have been described [41].

### Immunoblotting

Cells were washed with cold PBS and lysed in RIPA or Laemmli Sample buffer supplemented with β-mercaptoethanol, benzonase and MgCl_2_ (2 mM) (Biorad). Cell lysates were resolved by 4–20% or 7.5% SDS PAGE (Bio-Rad) and blotted onto nitrocellulose membrane using iBlot Dry Blotting System (Invitrogen). The following antibodies were used in this study: anti-FLCN [22], anti-FNIP1 [22], anti-β-actin (Sigma), anti-TFE3 (Santa Cruz, sc-5958), anti-GPNMB (R&D systems, AF2550), anti-GPNMB (Celldex Therapeutics, CR011) and anti-CREB (Cell Signaling, 9197) antibodies. Immunoblots were processed by the SuperSignal West Pico Chemiluminescent Detection System (Thermo Scientific) according to the manufacturer's protocols.

### RNA isolation, Quantitative real-time reverse transcription-PCR (RT-PCR), and microarray

Total RNAs were isolated from cells, tumors and kidney tissues using Trizol reagent (Invitrogen) and further purified using RNeasy mini kit (QIAGEN) according to the manufacturer's protocols. To confirm GPNMB expression, quantitative real-time reverse transcription PCR (RT-PCR) was performed as described [31]. Quantitative RT-PCR was performed with Power SYBR-Green or Taqman Gene Expression Master Mix (Applied Biosystems) using a 7300 Real-Time PCR system (Applied Biosystems) following the manufacturer's protocols. All reactions were run in triplicate using *β-actin*, *GAPDH* or *cyclophilin A* genes as internal controls. The gene-specific primer pairs for the PCR reactions are as follows: human *FLCN* forward 5′- TTCACGCCATTCCTACACCAGA -3′ and reverse 5′- GCCCACAGGTTGTCATCACTTG -3′, mouse *Flcn* forward 5′- ACCATGGAAGACAGCAAGCAT-3′ and reverse 5′- TGCACGTGACCTGAGAAGTCA-3′, human *GPNMB* forward 5′- TGCGAGATCACCCAGAACACA-3′ and reverse 5′-CGTCCCAGACCCATTGAAGGT-3′, mouse *Gpnmb* forward 5′-CCAGCCACTTCCTCAACGA-3′ and reverse 5′-CCAGTGTTGTCCCCAAAGTTC-3′, *ACP5* forward 5′-CGCTCCCTTCGCAAAGTG-3′; reverse 5′-TGCCAAGGTGGTCATGGTTT-3′; *ASAH1* forward 5′-GCGGCCTCTGAGACATGAAG-3′, reverse 5′-AGGTCAGACAGCTGCAGTGTTC-3′; *FNIP2* forward 5′-CTCACTTCTGGCGGGCTACT-3′, reverse 5′-GGTCCCATGAAGCACAAGATC-3′; *SULT1C2* forward 5′-CTGAGCGCCCCCGTAAC-3′, reverse 5′-GAAGCAGACGGCAATTGCA-3′. PCR product quality was monitored using post-PCR dissociation curve analysis. For expression microarray, probes were generated by 3′ *in vitro* transcription kit (Affymetrix) using total RNAs following their instruction. Human Genome U133 Plus 2.0 arrays (Affymetrix) were used for probe hybridization and processed according to recommended protocols. The CEL files were processed using the Partek Genomic Suite 6.4 (Partek Inc.). Data were transformed using a log normalization process and the differentially expressed genes were filtered using Microsoft Excel software.

### Immunohistochemistry

Renal tumors were resected from BHD patients and kidneys were collected from *Flcn*-heterozygous or kidney-specific conditional *Flcn* knockout mice [24], [26]. Five micron-thick, paraffin embedded tissue sections were treated with either proteinase K solution (DAKO RTU) at RT for 10 min for GPNMB or microwave heating in TE buffer (pH 8.0) for 20 min for TFE3 staining. After blocking, slides were incubated with biotinylated goat-anti-human GPNMB (R&D Systems, AF2550), biotinylated anti-GPNMB (Celldex Therapeutics, CR011) or with anti-TFE3 antibody (Sigma, HPA023881) for 30 min at room temperature at 1∶100 and 1∶500 dilutions respectively.

### Immunocytochemistry

Cells were cultured on chamber slides (LAB-TEK) and transduced with adenovirus expressing TFE3 (50× MOI). 24 hr after transduction, cells were fixed with 4% paraformaldehyde solution for 5 min and permeabilized with 0.3% Triton X-100. After washing three times with PBS and blocking with 5% BSA for 1 hr, cells were labeled with rabbit polyclonal anti-TFE3 antibody and then incubated with AlexaFluor 546 conjugated anti-rabbit antibody. Cells were covered with coverslips with Prolong Gold Antifade reagent (Invitrogen). Subcellular localization of TFE3 was examined under fluorescence microscope (Carl Zeiss).

### Cytoplasmic, and soluble and insoluble nuclear extracts preparation

Nuclear and cytoplasmic fractionation was performed basically following the protocol described by Andrews and Faller with minor modifications [43]. Cells were washed and resuspended in buffer A (10 mM HEPES-KOH pH 7.9, 1.5 mM MgCl_2_, 10 mM KCl, 0.5 mM DTT and 0.1% NP-40) containing Complete proteinase and PhosphoSTOP phosphatase inhibitor cocktails from Roche. After 10 min of swelling, cells were vortexed for 10 sec and centrifuged for 10 sec. The supernatant was used as the cytoplasmic fraction. The pellets were resuspended in buffer C (20 mM HEPES-KOH pH 7.9, 25% glycerol, 420 mM NaCl, 1.5 mM MgCl_2_, 0.2 mM EDTA and 0.5 mM DTT, 0.1% NP-40) containing proteinase and phosphatase inhibitor cocktails, incubated on ice for 20 min and centrifuged for 2 min. The supernatant was used as soluble nuclear extracts. The resulting pellets were resuspended in Laemmli Sample Buffer (Bio-Rad) containing benzonase and 2 mM MgCl_2_ and designated as insoluble nuclear extracts.

### Electrophoretic mobility shift assay (EMSA)

Nuclear extracts were isolated from UOK257 cells infected with adenovirus expressing TFE3 as described above. Probe DNAs were labeled with biotin using Biotin 3′ End DNA Labeling Kit from Thermo Scientific. Nuclear extracts were incubated with biotin-labeled probes for 30 min at room temperature and separated by electrophoresis through 6% DNA retardation gels from Invitrogen. Probe DNAs were detected using LightShift Chemiluminescent EMSA Kit (Thermo Scientific) following manufacturer's instructions. Probe and competitor DNAs used in EMSA included wildtype M box (5′-CCGCTTAATACCATCACATGATCCTCC-3′) and mutant M box (5′-CCGCTTAATACCATCTCGAGATCCTCC-3′). A nuclear factor 1 probe was used as a control (5′-TTTTGGATTGAAGCCAATATGATA-3′). TFE3/probe complex formation was competed with 200 fold excess amounts of unlabeled oligonucleotides containing wildtype M box or mutant M box. TFE3/probe band was super-shifted with anti-TFE3 antibody.

### Luciferase assay

*GPNMB* promoter reporter plasmid was described and kindly provided by N.A. Meadows [12]. Reporter plasmid DNAs were transfected into HT1080 cells along with TK promoter-driven renilla luciferase reporter plasmid (Promega, pGL4.74) using Lipofectamine 2000 reagent (Invitrogen). Cells were harvested 24 hrs after transfection and luciferase activity was measured using Dual-luciferase Reporter Assay system (Promega) following the manufacturer's instruction.

### Patient tumor samples, and care and use of animals

Approval was obtained from the NIH Institutional Review Board for this study. Written informed consent was obtained from patients for collection of human tumor tissue. All animal studies were conducted in an AAALAC-accredited facility, in compliance with the US Public Health Service guidelines for the care and use of animals in research under protocols (ASP#07-063) approved by the ACUC at the NCI-Frederick.

## Supporting Information

Figure S1 **FLCN expression is inversely correlated with GPNMB mRNA expression.** (A) Quantitative RT-PCR of GPNMB expression in the UOK257 cells expressing either wildtype or mutant FLCN. P, parental; HR, H255R. (B) FLCN knockdown induced GPNMB protein expression. (C) Adenovirus-mediated wild-type FLCN but not mutant FLCN (c.1285dupC) expression suppressed GPNMB protein expression. (D) Gpnmb mRNA expressions in *Flcn* heterozygous (B1, C1, D1 and D2) and *Flcn*-null (CA21, CA31 and DA21) MEFs. *f*, floxed allele; -, deleted allele. (E) Gpnmb mRNA expressions in the kidneys with *Flcn* inactivation by kidney specific Cre recombinase (Ksp-Cre) transgene expression.
(PDF)

Figure S2 **Nuclear localization of TFE3 in the** ***Flcn*** **-null MEFs.** (A) Cells were plated on chamber slides and TFE3 proteins were probed with rabbit polyclonal anti-TFE3 antibody (1∶450 dilutions) as described in Materials and Methods. Nuclei were counter-stained with DAPI. (B) Immunohistochemical staining of TFE3 in the renal tumors and adjacent normal kidney tissues from BHD patients, and in the alveolar soft part sarcoma.
(PDF)

Figure S3 **Regulation of ASAH1, ACP5, FNIP2 and SULT1C mRNA expression by FLCN and TFE3.** Quantitative RT-PCR of ASAH1, ACP5, FNIP2 and SULT1C2 expression after FLCN and/or TFE3 knockdown by siRNA in UOK257-2 cells.
(PDF)

Figure S4 **A highly conserved M-box sequence in the** ***FNIP2*** **ortholog promoters.** *FNIP2* ortholog promoter sequences were obtained from the Ensembl database (www.ensembl.org) and aligned with Clustalx (1.8) software.
(PDF)

Figure S5 **A model of regulation for TFE3 transcriptional activity by** ***FLCN*** **inactivation.** *FLCN* inactivation induces dephosphorylation and an additional post-translational modification of TFE3 that are necessary for its nuclear import and retention, and transcriptional activity. P, phosphorylation; PTM, post-translational modification; Enz, Enzyme; PPase, protein phosphatase.
(PDF)

Table S1 **The genes up- or down-regulated by FLCN expression in UOK257 cells.**
(PDF)

Table S2 **The list of genes induced by FLCN siRNAs and reduced by TFE3 siRNAs.**
(XLS)

Table S3 **The list of TFE3 regulated genes with MiTF/TFE binding sequence in their promoters.**
(XLS)
