# Supplementary material for: Inactivation of the FLCN Tumor Suppressor Gene Induces TFE3 Transcriptional Activity by Increasing Its Nuclear Localization
Source: PLoS One. 2010 Dec 29;5(12):e15793. doi: 10.1371/journal.pone.0015793 (PMC3012117; doi:10.1371/journal.pone.0015793)
Supplement: Figure S1 — FLCN expression is inversely correlated with GPNMB mRNA expression. (A) Quantitative RT-PCR of GPNMB expression in the UOK257 cells expressing either wildtype or mutant FLCN. P, parental; HR, H255R. (B) FLCN knockdown induced GPNMB protein expression. (C) Adenovirus-mediated wild-type FLCN but not mutant FLCN (c.1285dupC) expression suppressed GPNMB protein expression. (D) Gpnmb mRNA expressions in Flcn heterozygous (B1, C1, D1 and D2) and Flcn-null (CA21, CA31 and DA21) MEFs. f, floxed allele; -, deleted allele. (E) Gpnmb mRNA expressions in the kidneys with Flcn inactivation by kidney specific Cre recombinase (Ksp-Cre) transgene expression. (PDF) [file pone.0015793.s001.pdf]

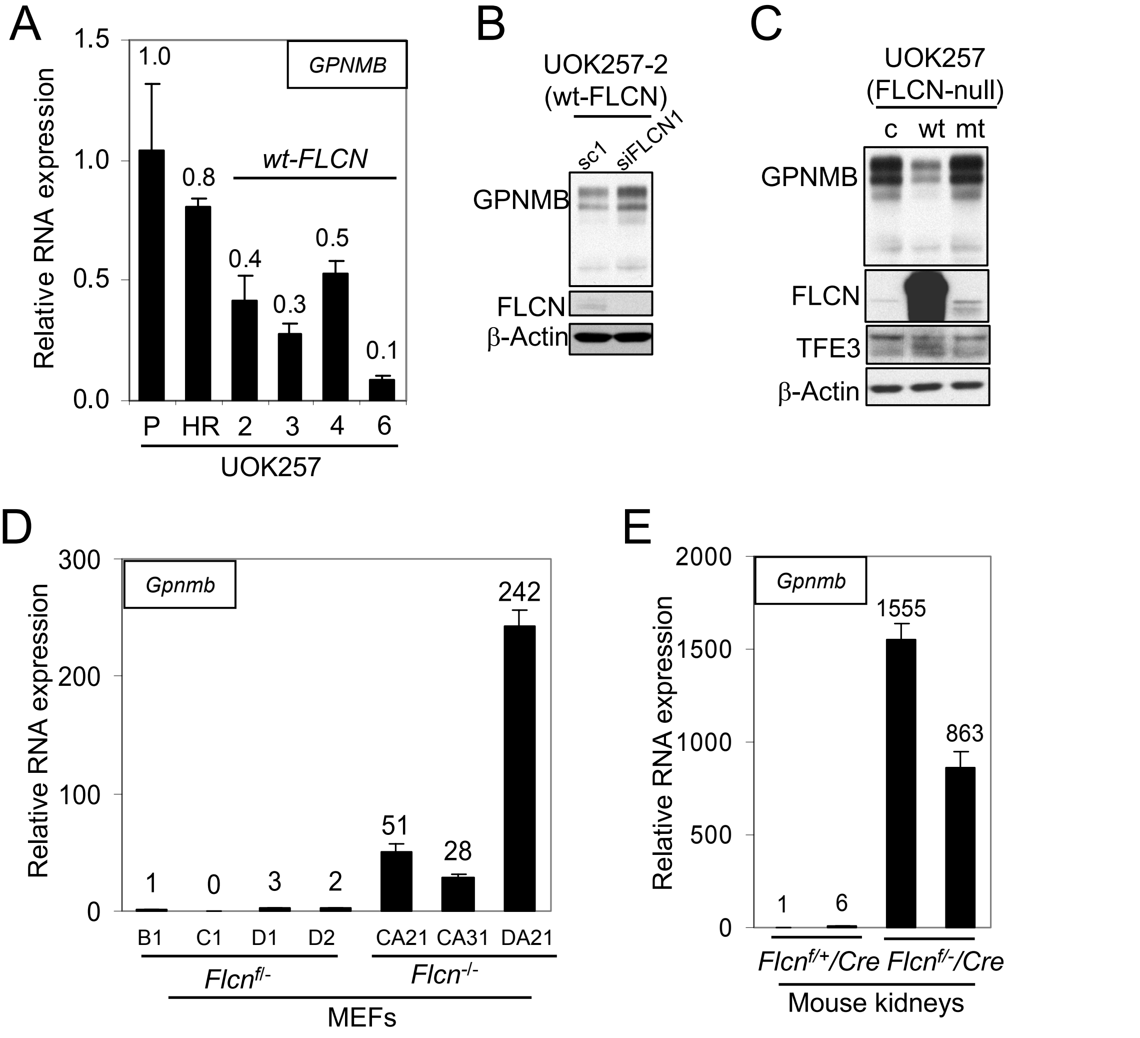

Figure S1. **FLCN expression is inversely correlated with *GPNMB* mRNA expression.** (A) Quantitative RT-PCR of GPNMB expression in the UOK257 cells expressing either wildtype or mutant FLCN. P, parental; HR, H255R. (B) FLCN knockdown induced GPNMB protein expression. (C) Adenovirus-mediated wild-type FLCN but not mutant FLCN (c.1285dupC) expression suppressed GPNMB protein expression. (D) Gpnmb mRNA expressions in *Flcn* heterozygous (B1, C1, D1 and D2) and *Flcn*-null (CA21, CA31 and DA21) MEFs. *f*, floxed allele; -, deleted allele. (E) *Gpnmb* mRNA expressions in the kidneys with *Flcn* inactivation by kidney specific Cre recombinase (Ksp-Cre) transgene expression.
