# Supplementary material for: Inactivation of the FLCN Tumor Suppressor Gene Induces TFE3 Transcriptional Activity by Increasing Its Nuclear Localization
Source: PLoS One. 2010 Dec 29;5(12):e15793. doi: 10.1371/journal.pone.0015793 (PMC3012117; doi:10.1371/journal.pone.0015793)
Supplement: Figure S2 — Nuclear localization of TFE3 in the Flcn -null MEFs. (A) Cells were plated on chamber slides and TFE3 proteins were probed with rabbit polyclonal anti-TFE3 antibody (1∶450 dilutions) as described in Materials and Methods. Nuclei were counter-stained with DAPI. (B) Immunohistochemical staining of TFE3 in the renal tumors and adjacent normal kidney tissues from BHD patients, and in the alveolar soft part sarcoma. (PDF) [file pone.0015793.s002.pdf]

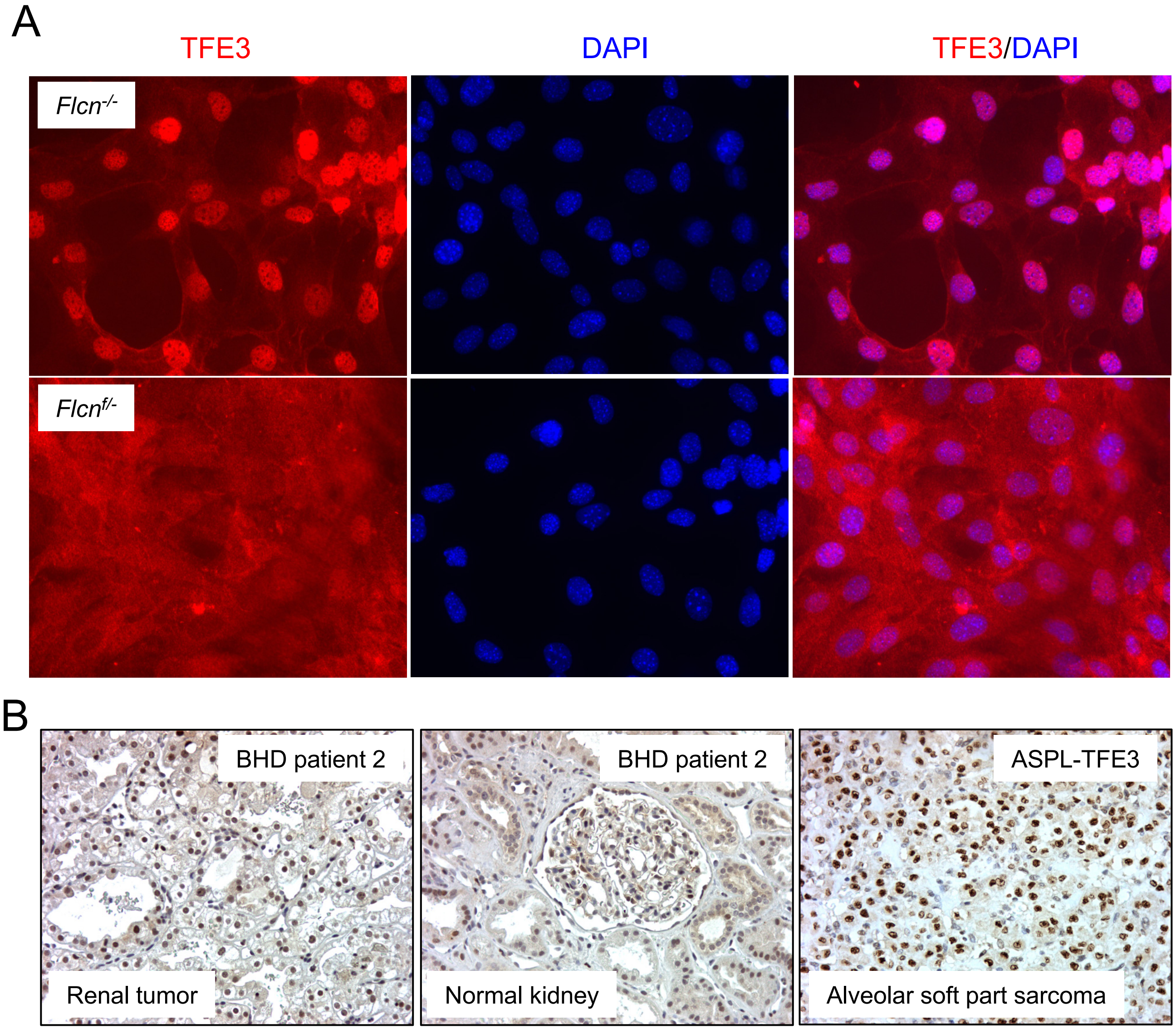

Figure S2. Nuclear localization of TFE3 in the *Flcn*-null MEFs. (A) Cells were plated on chamber slides and TFE3 proteins were probed with rabbit polyclonal anti-TFE3 antibody (1:450 dilutions) as described in Materials and Methods. Nuclei were counter-stained with DAPI. (B) Immunohistochemical staining of TFE3 in the renal tumors and adjacent normal kidney tissues from BHD patients, and in the alveolar soft part sarcoma.
