# Supplementary material for: Inactivation of the FLCN Tumor Suppressor Gene Induces TFE3 Transcriptional Activity by Increasing Its Nuclear Localization
Source: PLoS One. 2010 Dec 29;5(12):e15793. doi: 10.1371/journal.pone.0015793 (PMC3012117; doi:10.1371/journal.pone.0015793)
Supplement: Figure S3 — Regulation of ASAH1, ACP5, FNIP2 and SULT1C mRNA expression by FLCN and TFE3. Quantitative RT-PCR of ASAH1, ACP5, FNIP2 and SULT1C2 expression after FLCN and/or TFE3 knockdown by siRNA in UOK257-2 cells. (PDF) [file pone.0015793.s003.pdf]

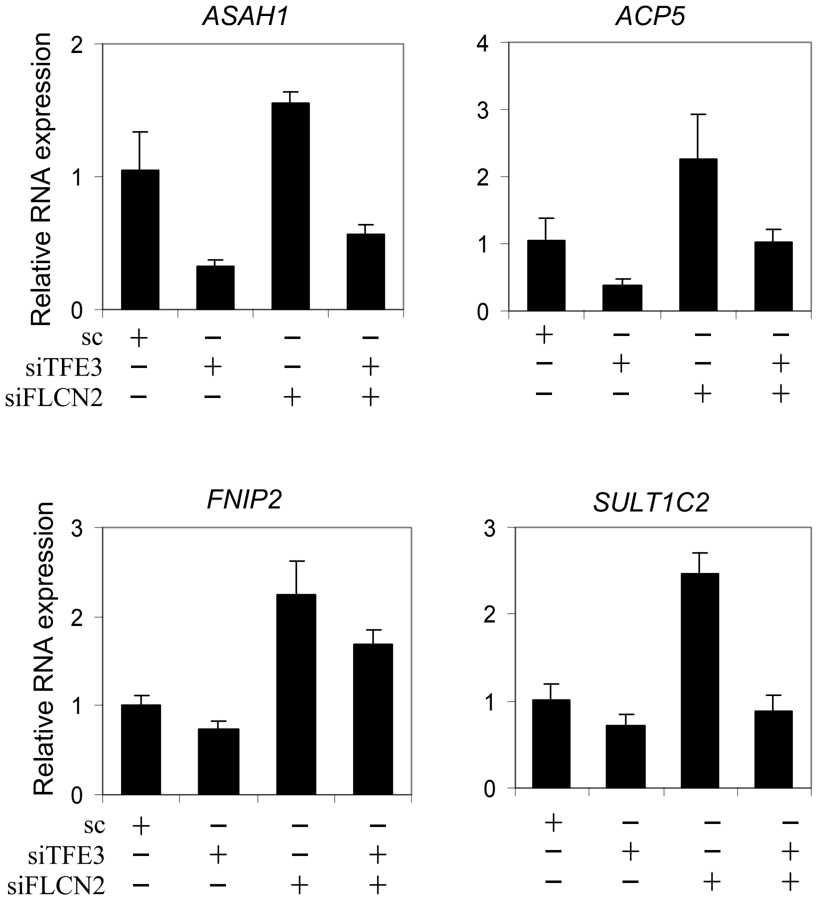

Figure S3. Regulation of ASAH1, ACP5, FNIP2 and SULT1C mRNA expression by FLCN and TFE3. Quantitative RT-PCR of ASAH1, ACP5, FNIP2 and SULT1C2 expression after FLCN and/or TFE3 knockdown by siRNAs in UOK257-2 cells.
