# Supplementary material for: Inactivation of the FLCN Tumor Suppressor Gene Induces TFE3 Transcriptional Activity by Increasing Its Nuclear Localization
Source: PLoS One. 2010 Dec 29;5(12):e15793. doi: 10.1371/journal.pone.0015793 (PMC3012117; doi:10.1371/journal.pone.0015793)
Supplement: Figure S4 — A highly conserved M-box sequence in the FNIP2 ortholog promoters. FNIP2 ortholog promoter sequences were obtained from the Ensembl database (www.ensembl.org) and aligned with Clustalx (1.8) software. (PDF) [file pone.0015793.s004.pdf]

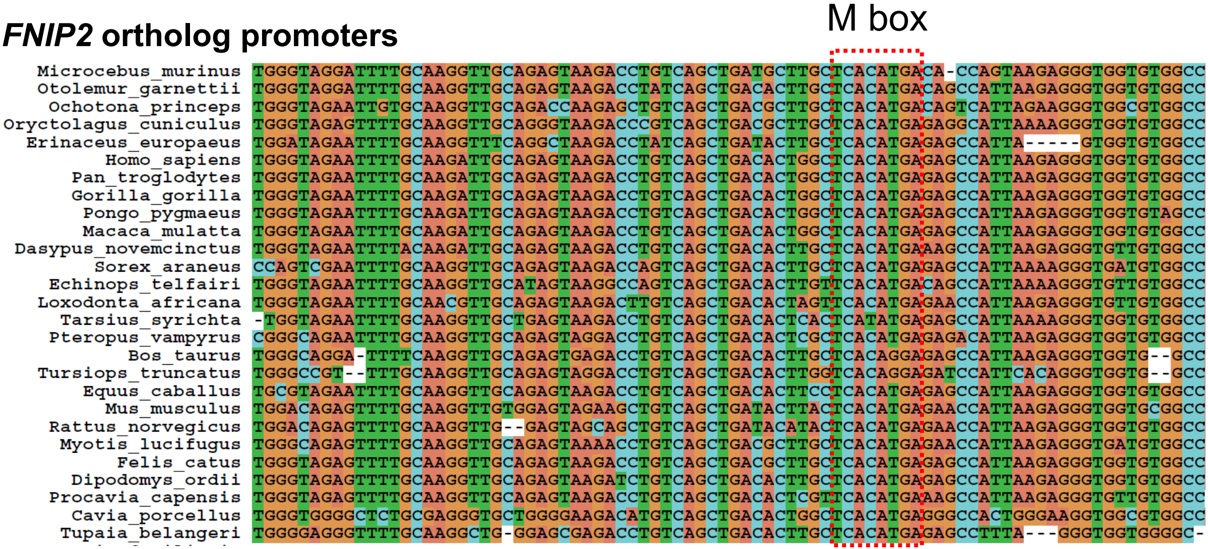

Figure S4. A highly conserved M-box sequence in the *FNIP2* ortholog promoters. Multiple sequence alignment shows a highly conserved M-box sequence in the *FNIP2* ortholog promoters. *FNIP2* ortholog promoter sequences were obtained from the Ensembl database (www.ensembl.org) and aligned with Clustalx (1.8) software.
