# Supplementary material for: Inactivation of the FLCN Tumor Suppressor Gene Induces TFE3 Transcriptional Activity by Increasing Its Nuclear Localization
Source: PLoS One. 2010 Dec 29;5(12):e15793. doi: 10.1371/journal.pone.0015793 (PMC3012117; doi:10.1371/journal.pone.0015793)
Supplement: Figure S5 — A model of regulation for TFE3 transcriptional activity by FLCN inactivation. FLCN inactivation induces dephosphorylation and an additional post-translational modification of TFE3 that are necessary for its nuclear import and retention, and transcriptional activity. P, phosphorylation; PTM, post-translational modification; Enz, Enzyme; PPase, protein phosphatase. (PDF) [file pone.0015793.s005.pdf]

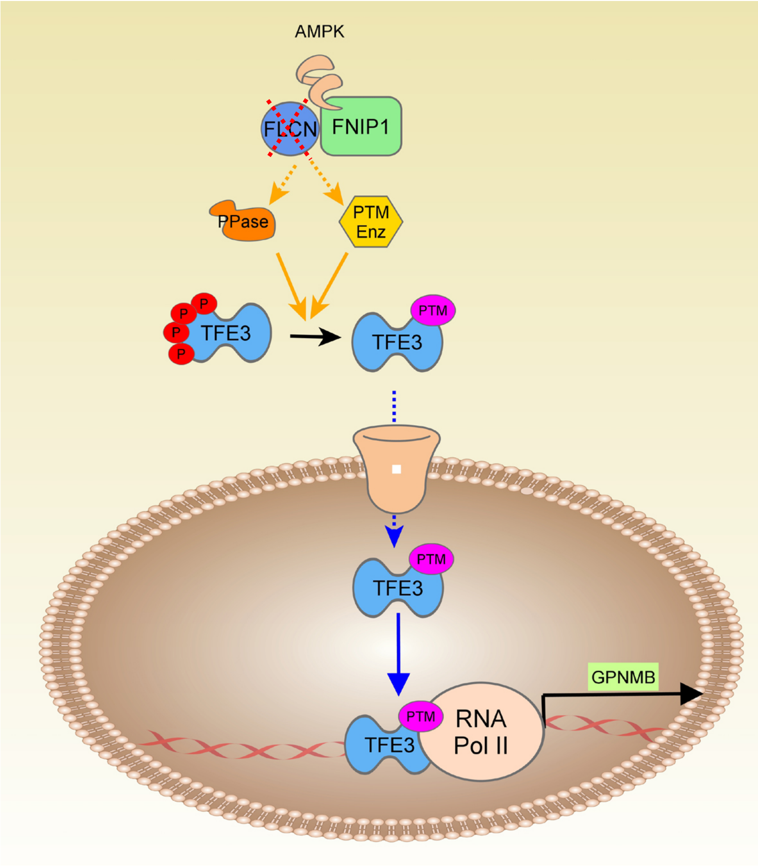

Figure S5. A model of regulation for TFE3 transcriptional activity by *FLCN* inactivation. *FLCN* inactivation induces dephosphorylation and an additional post-translational modification of TFE3 that are necessary for its nuclear import and retention, and transcriptional activity. P, phosphorylation; PTM, post-translational modification; Enz, Enzyme; PPase, protein phosphatase.
