# Supplementary material for: Inactivation of the FLCN Tumor Suppressor Gene Induces TFE3 Transcriptional Activity by Increasing Its Nuclear Localization
Source: PLoS One. 2010 Dec 29;5(12):e15793. doi: 10.1371/journal.pone.0015793 (PMC3012117; doi:10.1371/journal.pone.0015793)
Supplement: Table S1 — The genes up- or down-regulated by FLCN expression in UOK257 cells. (PDF) [file pone.0015793.s006.pdf]

Table S1. The genes up- or down-regulated by FLCN expression in UOK257 cells.

**Gene Symbol**	**Gene Name**	**Log_2 (*Fold)**
GPNMB	glycoprotein (transmembrane) nmb	-1.9
GREM1	Gremlin	-1.4
CDH1	cadherin 1, type 1, E-cadherin (epithelial)	-0.7
SMAD3	SMAD, mothers against DPP homolog 3	0.6
FYN	FYN oncogene related to SRC, FGR, YES	0.9
FABP3	Fatty acid binding protein 3, muscle and heart	0.9
MAL	mal, T-cell differentiation protein	1.1
TGFB2	Transforming growth factor-beta 2	1.1
DCDC2	doublecortin domain containing 2	1.5
VCAM1	vascular cell adhesion molecule 1	1.6
THBS1	thrombospondin 1	1.8
SOX9	SRY (sex determining region Y)-box 9	2.2
INHBA	inhibin, beta A	2.5
CDH13	Cadherin 13, H-cadherin (heart)	2.9
TNC	tenascin C (hexabrachion)	4.4

*, The level of gene expression induced or reduced by Ad-FLCN was divided by the level of gene expression by Ad-LacZ.
